# Training nurses to facilitate and implement CURA in palliative care institutions: development and evaluation of a blended learning program

**DOI:** 10.1186/s12904-023-01284-4

**Published:** 2023-10-21

**Authors:** Malene van Schaik, Charlotte Kröger, Lisa Zuidema, Margreet Stolper, Guy Widdershoven, H. Roeline Pasman, Suzanne Metselaar

**Affiliations:** 1 Department of Ethics, Law and Humanities, Amsterdam UMC Location VUmc, Amsterdam, The Netherlands; 2 Department of Public and Occupational Health, Expertise Center for Palliative Care, Amsterdam UMC Location VUmc, Amsterdam, The Netherlands

**Keywords:** Clinical ethics support, CURA, Palliative care, Ethics education, Evaluation, Blended learning

## Abstract

**Background:**

Healthcare professionals in palliative care are found to be confronted with moral challenges on a frequent basis. CURA is a low-threshold instrument for dialogical ethical reflection that was developed to deal with these challenges. A previous study identified the need of healthcare professionals to be trained to introduce CURA in their organization, initiate and facilitate reflections with CURA, and contribute to the implementation of CURA. The aim of this study was to develop and evaluate a training for professionals to become ‘CURA-ambassadors’.

**Methods:**

The training was developed in a participatory way in two cycles. We trained 72 healthcare professionals. The training was evaluated by means of a questionnaire and six semi-structured interviews.

**Results:**

The study resulted in a blended learning training combining training sessions with an e-module and with practicing with organizing and facilitating CURA in daily healthcare practice. The main objectives of the training are to enable CURA-ambassadors to introduce CURA within their organization, initiate and facilitate ethical reflections using CURA, and contribute to the implementation of CURA. Participants were generally positive about the training program and the trainers. Technical difficulties related to the e-module were mentioned as main point of improvement.

**Discussion:**

The training program can generate ownership, responsibility, and competency among CURA-ambassadors, which are essential foundations for implementing complex interventions in healthcare practice. The training program received positive evaluations shortly after completing the program. This study adds to our understanding of what is needed for healthcare professionals to use CURA, in order to support them in dealing with moral challenges and to foster their moral resilience. Further research is needed to assess whether participants experience the training as sufficient and effective when using and implementing CURA structurally in their organizations over a longer period of time.

**Supplementary Information:**

The online version contains supplementary material available at 10.1186/s12904-023-01284-4.

## Introduction

Healthcare professionals in palliative care are often confronted with moral challenges, leading to high levels of ‘moral distress’: the psychological distress that is causally related to a moral event [1]. Experiencing moral distress can cause burn out, personnel shortages due to sick leave and turnover rates [2–4]. Furthermore, it can impact the quality of care provided [5]. One proposed manner to mitigate moral distress, is by fostering ‘moral resilience’: the capacity to sustain or restore one’s integrity in response to moral adversity [6]. We have developed a Clinical Ethics Support instrument called CURA to support healthcare professionals in dealing with moral challenges in daily practice by fostering their moral resilience and to support them in providing quality care [7].

**Fig. 1:**
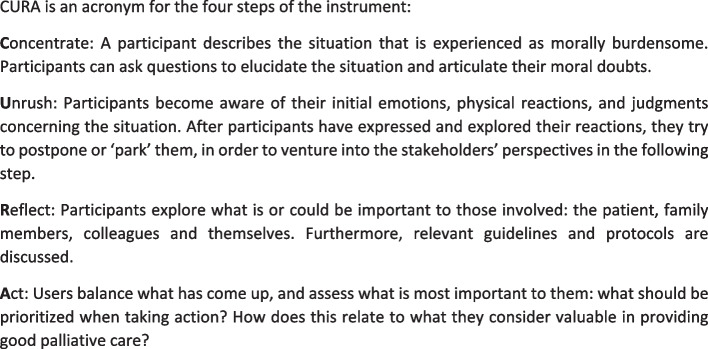
The four steps of CURA. The instrument is described in detail elsewhere [7]

CURA is a low threshold instrument for ethical reflection consisting of four main steps (see Fig. 1). It is designed to be used by healthcare professionals in a timeframe of approximately 40 min, either individually or in a small group, to reflect on a concrete ethical issue that is experienced in practice. CURA was co-created with various stakeholders in a participatory development study in the context of Dutch palliative care [8]. CURA is primarily developed for nurses and nurse assistants but can also be used by other healthcare professionals and in heterogeneous groups. CURA was designed to be used without extensive preparatory training, in order to lower the threshold for methodically structured ethical reflection on moral dilemmas in daily practice [7]. In order to support healthcare professionals in working with CURA, a handout, manual, and introductory workshops were made available. However, in a feasibility study [9], healthcare professionals expressed the need for extra support, especially in situations in which they were to introduce and implement CURA in their organization or team. Therefore, we developed a blended learning training program for ‘CURA-ambassadors’. These ‘CURA-ambassadors’ are healthcare professionals that have completed the training and are the promoters of CURA in their organization. The training prepares them for 1) introducing CURA within their organization, 2) initiating and facilitating reflections in small groups with colleagues using CURA to reflect on moral challenges in their practice, 3) contribute to the implementation of CURA within their own organization.

The development of this training was part of a three-year implementation study (2020- 2023) in the Netherlands, in which 10 healthcare organizations participated. In the Netherlands, palliative care is provided mainly by generalist healthcare professionals, and if needed by specialists [10]. Patients with palliative care needs are found in diverse healthcare settings, i.e.: hospices, hospitals, home care and nursing homes. Therefore, our aim was to develop a training that effectively trains healthcare professionals in all these settings. This paper describes the content and evaluation of the training for CURA-ambassadors.

## Method

### Development of the training

We have developed the training using a participatory development design [11]. In this design, end-users and other stakeholders are closely involved throughout every step of the development of the training. We have involved our stakeholders by forming a Community of Practice (CoP); a group of experts sharing experiences and information, that interacts regularly to reach a shared goal and to learn from and with each other [12]. Our CoP (*n* = 8) convened online three times with six months intervals to discuss the aims, content, and evaluation of the training program. The CoP consisted of an expert of implementation in the field of palliative care, a nurse working in palliative care, experts in training and education in both palliative care and clinical ethics, and a technical developer of e-modules.

A first concept of the learning goals and content of the training was made on the basis of our findings from a feasibility study on CURA [9]. This provided us with first insights into what healthcare professionals would need in order to initiate and use CURA in practice. We also built on the format of an existing training program for facilitators of Moral Case Deliberation, a clinical ethics support (CES) instrument that bears similarities with CURA [13]. Subsequently, this concept was discussed with our CoP. Based on the input of our CoP, we developed the first version of the training.

Subsequently, we piloted, evaluated and refined the training in two cycles. In the first cycle, 46 trainees from seven health care organizations participated. The organizations received governmental funding to participate in this study. At the end of the first training cycle, we evaluated the training by means of a questionnaire among the participants and the observational notes made by the trainers. We discussed the findings of our evaluation in the second session with our CoP. On the basis of this discussion, we refined the training program. In the second cycle, the training was offered to 25 trainees from three other organizations. These participants were also invited to evaluate the training by means of a questionnaire.

### Evaluation of the training

#### Data collection

**Fig. 2:**
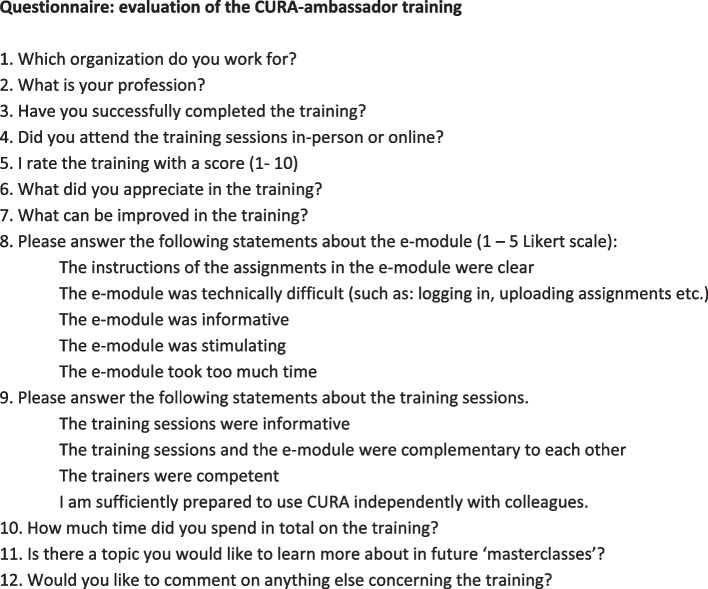
Items questionnaire for evaluation of the training program

All participants who participated in the three training sessions were invited to fill out the online questionnaire. We included those participants who did not complete the e-learning part of training, or did not practice in between meetings, in order to decrease the possibility of selection bias. Furthermore, their response might generate valuable insights into their wishes and needs, and why they did not complete the other parts of the training. The questionnaire was developed specifically for this study and consisted of items with a 5-point Likert scale and open-ended questions (see Fig. 2, ans as Supplementary file). The questionnaire was sent via Suvalyzer after the training was finished.

**Fig. 3:**
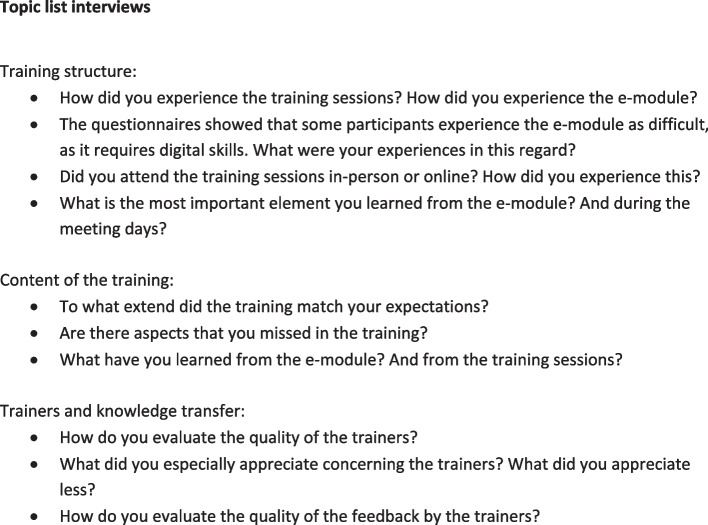
Topic list for semi-structured interviews

The results of the questionnaire were triangulated using six in-depth semi-structured interviews. The topic list is depicted in Fig. 3. Participants for the interviews were selected using purposeful sampling. We aimed to generate a maximum variation to include a broad range of perspectives. We have purposefully recruited participants that had different professions and/or worked for different healthcare organizations and settings. We included participants that had followed the training sessions either live or online. Interviews took 20 – 30 min and were audio recorded and transcribed verbatim.

#### Analysis

Analysis of the questionnaires was done using descriptive statistics in SPSS 26.0. We analyzed the data after the first cycle. We used these insights to inform our CoP-members and to make adjustments to the training program before starting the second cycle. Analysis of the second set of questionnaires was performed after the second cycle. Thematic analysis of the interviews was done using MaxQDA 22.0. We used a deductive approach, taking the results of the quantitative analysis as start. The codes were discussed in the research group and analysis was done by two researchers (L.Z. and M.V.S.).

## Results

In this section, we will describe the content and evaluation of the training.

### Content

- **Learning objectives**

**Fig. 4:**
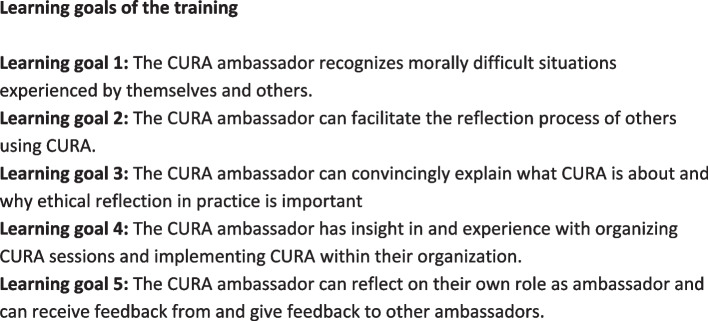
Main learning goals of the training

On the basis of co-creation with our CoP, we formulated five main learning goals of the training (Fig. 4), focusing on development of competencies that are needed to initiate, facilitate, and participate in implementing ethical reflection with CURA within an organization.

b.  **Structure**

We chose a blended learning approach to our the training. Blended learning, i.e., the combination of training sessions or instructions and technology-mediated instructions or programs [14–16] holds several advantages. First, it gave us the opportunity to optimally utilize the training sessions to practice the facilitation of CURA and to discuss implementation, while transferring knowledge via the e-module. Second, participants could have more control over their learning trajectory and follow the technology-mediated part of the training at their own pace [17]. This is convenient for healthcare professionals that work at different locations and have varying work schedules. Third, the e-module platform enabled the participants to interact with each other. Finally, the platform enabled them to upload materials that helped the trainers to track and evaluate their progress.

**Fig. 5:**
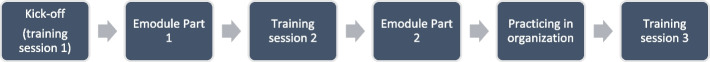
Structure of the training program

The training consists of three elements: (1) an e-module, (2) three training sessions (one-kick-off meeting and two meetings to practice the facilitation of CURA and to discuss implementation), and (3) practicing with organizing and facilitating CURA sessions within their own organization (see Fig. 5). Four to five weeks are given in between training sessions, giving participants time to complete the e-module and to practice organizing and facilitating CURA in their own work environment. The entire training program takes approximately 23 h to complete. The content of the training is as follows:

*Kick-off:* An introductory kick-off meeting of 75 min, focusing on becoming acquainted with each other, CURA, and the set-up of the training. In order to introduce CURA, the trainer facilitates a CURA session using a case of one of the participants. Furthermore, couples are set up using the ‘buddy system’. Buddies will give each other feedback on the assignments of the e-module and serve as sparring partners throughout the training.

*E-module part 1:* Participants can individually go through the e-module at their own pace, either at work or at home. The content consists of reading material (such as the CURA manual for facilitators and an e-book with more background information on the instrument); two videos with clarification of CURA and background information on moral distress and moral resilience [1, 6]; and several assignments such as an interactive demo of using CURA with a pre-given case.

*Training session 2 and 3:* each of these meetings lasts three hours. All participants facilitate at least one CURA-session based on a case presented by one of the group members, under supervision of a trainer during the meeting. The participants receive feedback from the trainers and other participants on their performance as facilitators. Furthermore, there is time for sharing experiences and questions regarding the e-module. One or two trainers are present, depending on the group size.

*E-module part 2:* The second part of the e-module is done in between the second and the third training sessions. This e-module focuses primarily on the dissemination and implementation of CURA. Participants are instructed to prepare and deliver a pitch for their colleagues in order to raise publicity for CURA. This could either be a presentation, a video or a newsletter. Furthermore, they get a short introduction on how to write an implementation plan and then are tasked with writing their own 2-page plan. In this plan, they envision what successful implementation of CURA entails and what they need to achieve this. They distinguish promoting factors from barriers for implementation and what their own role in the implementation of CURA could look like. Next, the buddy-couples read each other’s implementation plan and discuss them. They document what they learned from each other’s plans. If necessary, they can adjust their implementation plan. The implementation plans are discussed during the second training day. Furthermore, the plans are used as an inspiration to write a more elaborate implementation plan after the training is completed (described in another paper).

*Practicing with colleagues:* in between the second and the third training sessions, participants practice their role as a facilitator at least twice using CURA in a small group setting with colleagues. Depending on their working environment and preference, they are able to choose with whom, when, and where they organize and facilitate a CURA session. Their buddy is present to observe and give feedback afterwards using a standardized handout. Participants also fill out self-assessment forms to stimulate reflection on their skills and development.

At the end of the training program, participants receive a certificate if they have successfully completed the entire program. They also receive an evaluation with personal feedback from one of the trainers.

The overall structure and content of the training is presented in Table 1.

**Table 1:** Content and structure of the training program

Structure of the training program		
Training element	**Description**	**Duration**
Kick off meeting:	Group session. Getting acquainted with each other and CURA. Using CURA for the first time as a participant	1 h
E-module part 1	- Two videos about CURA and its aims; introduction to theoretical concepts such as moral distress and moral resilience - Reading material on CURA and background information - Keeping a logbook of moral challenges, to develop moral sensitivity - Interactive demo of CURA - How to use CURA individually - Formulating personal learning objectives - Quiz - Discussing e-module with a peer	7 h
Training session 2	In-person or online. One or two trainers, depending on group size - Practicing with CURA and the role of facilitator. Cases are brought forward by participants. Facilitators receive feedback from each other and from the trainer - Questions that emerged from the e-module are discussed	3 h
e-module part 2	- A podcast on a nurse’s experience with palliative care and an emotional burdensome event - Preparing and delivering a pitch for colleagues - Following a short introduction to writing an implementation plan - Writing an implementation plan and discussing it with buddy	4 h
Practicing CURA with colleagues and observing as buddy	- Participants practice as a facilitator at least two times with colleagues (> 2). Reflect on their skills using a self-assessment form and receive feedback form from buddy - Observing CURA moments and giving feedback to buddy	5 h (including organizing CURA moment and debriefing in couples)
Training session 3	In-person or online. One or two trainers - Practicing with CURA and the role of facilitator - Discussing implementation and dissemination of CURA in organization - Reflecting on participants’ learning objective - Closing of the training program	3 h

### Evaluation

In this section, we present the findings of the evaluation of the training program of both cycle 1 and 2. We will first present the characteristics of the participants in the training, and the setting.

- **Characteristics and setting**

Participants in the CURA-ambassador training were recruited by the participating healthcare organizations. Characteristics of participants are presented in Table 2.

**Table 2:** Characteristics of ‘CURA-ambassadors’

	**Training participants (*n* = 72)**	**Questionnaire (*n* = 41)**
**Variable**	**n/ %**	
Age	*	
< 30	10 (15%)	
31- 40	14 (21%)	
41- 50	16 (24%)	
51- 60	19 (28%)	
> 60	8 (12%)	
Years of work experience	**	
0 – 5	28 (45%)	
6- 10	9 (19%)	
11- 20	15 (24%)	
> 21	10 (16%)	
Female	63 (88%)	
Setting	***	
- Home care	12 (17%)	9 (22%)
- Nursing home	28 (40%)	15 (37%)
- Hospital	23 (33%)	12 (29%)
- Hospice	7 (10%)	5 (12%)
Profession		
- LPN and HC assistant	21 (29%)	9 (22%)
- RN	19 (26%)	14 (34%)
- Spiritual counselor	8 (11%)	7 (17%)
- Nurse with specialized expertise in palliative care	7 (10%)	2 (5%)
- Other	17 (21%)	9 (22%)
Completed the training program	65 (92%)	36 (88%)

^*^missing: 10

^**^ missing: 5

^***^ missing: 2

Forty-one participants filled out the questionnaire (first cycle: *n* = 28 / response rate: 61%), second cycle: *n* = 13/ 52%). The overall response rate was 57%. Most participants were registered nurses (14/ 34%); followed by licensed nurse practitioners / health care assistants (9 / 22%) and spiritual counselors (7/ 17%). 2 (5%) were physicians; 2 (5%) were physician assistants and 7 (17%) had a different profession. Furthermore, six interviews were held after all organizations were trained in order to deepen our insights into the quantitative data from the questionnaires. These participants worked in nursing homes (*n* = 2), hospitals (*n* = 1), hospices (*n* = 1), and in home care (*n* = 2). Participants were nurses (*n* = 2), nurses with a specialization in palliative care (*n* = 2), physicians (*n* = 1) or spiritual counselors (*n* = 1).

Due to COVID-19 restrictions, not all training sessions were in-person; most were online. We followed the organization’s guidelines and national restrictions that were effective at the time. Most participants followed the training sessions online (24 / 59%). 11 (27%) followed the meetings in-person, and 6 (15%) had a mix of in-person and online meetings. The mean time spent on the entire training program was 23 h (SD: 9.7).

b. **Findings**

Overall, most participants evaluated the training program positively. The training as a whole received a mean score of 8 on a 10-point scale (range: 7 – 9). Results of the items measured with a 5-point Likert scale are presented in Table 3.

**Table 3:** Results of questionnaire items (*n* = 41)

	**Strongly disagree**	**Disagree**	**Neutral**	**Agree**	**Strongly agree**
The instructions of assignments in the e-module were clear	0	0	3 (7%)	29 (71%)	9 (22%)
The e-module was technically **difficult** (such as: logging in, uploading assignments etc.)	3 (7%)	12 (29%)	12 (29%)	13 (32%)	1 (2%)
The e-module was instructive	0	0	0	32 (78%)	9 (22%)
The e-module was stimulating	0	1 (2%)	9 (22%)	25 (61%)	6 (15%)
The e-module took **too much** time	3 (7%)	26 (63%)	10 (24%)	2 (5%)	0
The training sessions were instructive	0	0	1 (2%)	20 (49%)	20 (49%)
The training sessions and the e-module were complementary to each other	0	0	2 (5%)	24 (59%)	15 (37%)
The trainers were competent	0	0	0	11 (27%)	30 (73%)
I am sufficiently prepared to use CURA independently with colleagues	0	0	2 (5%)	27 (66%)	12 (29%)

Our qualitative data, both from the questionnaire and the interviews, can be subsumed in four topics: content of the training, training sessions (virtual or in-person), e-module, and acquired competencies relevant to being a CURA-ambassador.

### Content of the training

In general, the training was positively evaluated, and the content was experienced as relevant and instructive. Participants were especially positive about the competences of the trainers (73% strongly agreed). On the open-ended question ‘what did you appreciate about the training?’, some participants responded that they especially appreciated the fact that they had learned new skills, such as being able to better recognize and deal with moral challenges. Furthermore, they mentioned that the theoretical foundations were clearly explained and well connected to examples from daily practice. Other participants responded that they appreciated the variety in the training program: *“the training is well put together. It focuses on the content of CURA; the use of CURA in practice (implementation and how to deal with obstacles) and on facilitation techniques.”* [Questionnaire].

While participants were positive of the content, the open-ended questions and the interviews showed that participants were still in need of more knowledge about specific elements of CURA and implementation. One participant wrote: *“How to cope with resistance from colleagues? And I would like to learn how to deepen insight in the step Unrush during the CURA-process.”* [Questionnaire].

### Training sessions

Participants appreciated the open and safe environment and the personal feedback they received from the trainers. One of the participants of the interviews mentioned: *“it is a bit uncomfortable at first [to facilitate]. But the trainers knew how to ensure a safe space. They gave very good feedback, but also a lot of compliments, which made it a very nice experience for me.”* [Interview R1, Physician].

Participants appreciated learning from and with each other during the training sessions: *“The sessions were meaningful. I learned the most from practicing together”* [Questionnaire]. Due to COVID-19 restrictions, most training sessions were online. This was mentioned as a potential improvement for the training. As one of the participants mentioned in the open-ended questions of the questionnaire: *“It was due to the circumstances [COVID-19], but I prefer in-person meetings*.” [Questionnaire].

### E-module

The e-module was appreciated in general. One participant wrote: *“E-module: Short and to-the-point. (…) The variety of videos, podcast, and text was nice*.” [Questionnaire]. During the interviews, one participant mentioned that she noticed some overlap in the online material, for instance in the videos and the manual. She appreciated the overlap—because it can be useful to repeat important elements – and she also liked that she could go through certain elements more quickly: *“That’s the benefit of online: you can just scroll through it. You put your own twist on it.”* [Interview R3, nurse].

However, a third (35%) experienced technical difficulties with the e-module. For instance, they experienced difficulties in uploading assignments: *“For me the e-module was an obstacle. It might have helped if there was more instruction available.”* [Questionnaire]. Based on these findings, we resolved the technical problems by improving the structure of the e-module: we changed instructions that were perceived as confusing, improved the lay-out, and added extra instructions to create more lucidity. For instance, we wrote instructions on how to use the online environment. These instructions appeared upon the first entrance of the e-module, ensuring that all participants were informed. Participants were positive about the length and the content of the e-module; therefore we did not make adjustments in those respects.

### Competence in using CURA

The training is aimed at learning CURA-ambassadors to initiate and facilitate CURA sessions with colleagues in a small group setting. Hence, we asked participants whether they felt confident to do so. Most participants (95%) on the questionnaire (strongly) agreed with the item “I am sufficiently prepared to use CURA independently with colleagues”. However, during the interviews, one participant mentioned that she did not feel fully equipped to implement CURA by herself: *“It would’ve been nice if [the trainer] would reach out to us more [after completion of the training]. So, we can say ‘we struggle with this or that’, and perhaps we could get some advice”* [Interview R6, registered nurse].

Furthermore, support from others in the organization, specifically from managers, was considered essential for successful implementation: *“[Another CURA-ambassador] wanted to implement it in her team but her manager thought it would cost too much time and money. She really had to give good arguments and needed support from the CURA-project leader before she could start.”* [Interview R4, nurse].

## Discussion

This study aimed to describe the content and evaluation of the training for ‘CURA-ambassadors’ in detail. The goal of CURA is to provide clinical ethics support to healthcare professionals working in palliative care. We have developed the training program especially for healthcare professionals who seek to introduce, facilitate and implement CURA in their organizations. This study aims to describe how the training program was developed as well as its evaluation. This adds to our understanding of what is needed for healthcare professionals to be CURA ambassadors in their organization and, in this capacity, develop ethics support structures based on CURA. Furthermore, it provides insight into how to evaluate similar ethical training programs. We will discuss both the content and the evaluation of the training below.

### Content of the training

The content of the training is in line with an approach to clinical ethics support that focuses on context-based experiences and an approach to ethics training based on ‘learning by doing’ 7 13 [18]. Rather than focusing on abstract ethical theory or theoretical cases only, the training centralizes exercises based on the actual experiences and moral challenges of the participants. It provides ample room for practicing as a facilitator under the supervision of a trainer. The e-module consists of information about the content on CURA and (interactive) exercises to learn to use and implement it within their organization.

Training healthcare professionals as CURA-ambassadors can generate ownership, responsibility, and competency in introducing, facilitating, and implementing the instrument. All of these aspects are essential factors for implementing complex interventions [19–22]. Furthermore, training programs aimed at strengthening the moral competences of HCPs may improve the quality of palliative care to patients and their families [23].

The selection of the right individuals is imperative for successful implementation [24]. These individuals are the ‘driving forces’ that enable change: they should have the ability to motivate others and communicate a vision. In this study, the researchers had limited influence on the selection process, as the healthcare organizations themselves selected the CURA-ambassadors. We suggested selecting participants that had affinity with reflection on moral issues, were working in palliative care, and intended to work at the organization for a longer period of time as to ensure sustainable commitment as a CURA-ambassador. One of the lessons learned from this study, is the importance of selecting CURA-ambassadors that also possess skills such as communication and motivational skills.

However, CURA-ambassadors alone cannot be held responsible for successful implementation: they need to be supported by their organization [19, 21, 22]. Some CURA-ambassadors experienced barriers when introducing CURA in their team, for instance due to a lack of support from management, or because the structures were not in place to implement CURA. For instance, in one home care organization, there were no team meetings at all: leaving no option for colleagues to meet each other on a regular basis. Hence, successful implementation requires commitment from all layers of the organization [25] and certain conditions need to be in place, such as enough time for reflection and a culture which fosters communication between colleagues [26]. An implementation study on the barriers and facilitators for implementing CURA will be published in the near future.

### Evaluation of the training

We have evaluated the training for CURA-ambassadors using a questionnaire (*n* = 41) and six semi-structured interviews with participants. The results of our questionnaire and interviews are divided into four themes: (1) content of the training, (2) face-to-face training sessions, (3) e-module, and (4) competencies that were trained. Our results show that participants were appreciative of being able to practice as a facilitator under the supervision of a trainer during the training sessions, and of the practical and interactive nature of the training, in which they could reflect on moral dilemmas from their own clinical setting. Participants remarked that they felt safe to practice their facilitation techniques, which has been identified as a key aspect for learning ethical skills [27].

Furthermore, participants were appreciative of the structure of the blended learning, enabling them to go through the e-module at their own pace and time, while the training sessions focused on practicing with CURA, receiving detailed feedback, and discussing implementation of CURA. However, it is important to be aware of digital inequality and/or digital illiteracy and to provide adequate support [28, 29]. Not all participants felt confident working with the e-module. This was due to some technical difficulties and errors in the first version of the e-module, and because some participants did not have any prior experience with e-modules. Some indicated they hardly used computers. Between cycle 1 and 2 we made the e-module more user-friendly; we gave more technical instructions, and we asked every organization to appoint one of the participants with technical skills or preliminary experience with e-modules to assist their peers if they stumbled upon difficulties while using the e-module. The comments on technical difficulties were only found in the evaluation after cycle 1, and not after cycle 2. This indicates the technical difficulties were addressed effectively.

The fourth theme was competence in using CURA. Most participants stated they felt sufficiently prepared to use CURA independently with colleagues. However, during the interviews, that took place a couple of months after completing the training, some participants expressed the need for more support from the trainers, as they experienced some difficulties with facilitating and/or implementing CURA. Apparently, right after the training participants felt confident in using CURA, but experienced difficulties in practice later on. This dynamic has also been described elsewhere [19, 21].

We would like to highlight two ‘lessons learned’ from this study. First, while it is important that organizations allocate resources for implementing an ethics support intervention [30], our participants also expressed the need for trainers to remain involved in the implementation phase and to stay connected with the participants after completion of the training. Therefore, we started to organize masterclasses for trained CURA-ambassadors in order to advance their expertise. The themes of these masterclasses are determined based on what CURA ambassadors themselves indicate they need. This could be specific facilitation skills or discussing experiences with implementation of CURA in various organizational and professional contexts.

Second, evaluation studies on ethics training programs often take place directly after completion of the training [23, 31–33] or not at all [19]. Furthermore, these studies, similar to this study, often of the training. A research design using a pre-post design and ideally a control group and following the participants over a longer period of time, will lead to more insights into the effectiveness of the training.

### Strengths and limitations

A strength of our study is that we developed and evaluated the training in a participatory way, i.e. in close collaboration with stakeholders, such as end users, experts in palliative care, education, e-learning, and/or ethics training. This participatory approach promotes meeting the wishes, preferences, and needs of end users [29]. Another strength is that the development process was iterative; we used the experiences of participants to improve the training.

This study has some limitations. First, as already described, the response rate of our questionnaire was relatively low (57%). This may be due to the fact that participants were still in the midst of dealing with the COVID-19 pandemic at that time, and might have been too occupied to complete questionnaires. The response rate varied substantially between organizations. For instance, in one home care organization, only one participant filled out the questionnaire. We have tried to overcome this limitation by including a participant from this organization in the interviews, ensuring their perspective was taken into account.

A second limitation is that the evaluation, consisting of the questionnaire and interviews, was conducted relatively shortly—within two months – after completion of the training. It might have been too early for participants to assess whether they were indeed sufficiently prepared as CURA ambassadors. In the period after this study was conducted, some participants indeed indicated they still could use more training, supervision and peer support. Hence, following the participants for a longer period of time could help meet their needs, establish successful implementation, and adjust the training module accordingly for future participants. Furthermore, we halve only conducted six interviews. Including more respondents could have provided us with more insight in the various experiences of end-users.

## Conclusions

This study adds to our understanding of what is needed by healthcare professionals in order to be ‘CURA-ambassadors’, i.e., healthcare professionals who are to introduce CURA, an ethics support instrument, in their organization, to initiate and facilitate ethical reflection with CURA, and to contribute to its implementation. This study provides insight into both the content and the evaluation of a blended learning training for CURA ambassadors. The training was co-created together with stakeholders in palliative care and evaluated using both questionnaires and interviews, shortly after the participants finished the training. Whereas the first evaluations were positive, follow-up research is needed to assess whether participants experience the training as sufficient and effective when using and implementing CURA structurally in their organizations.

### Supplementary Information

**Additional file 1. **

**Additional file 2. **

## Data Availability

The datasets generated and analyzed during the current study are available from the corresponding author on reasonable request. The CURA handout and manual are available free of charge from the corresponding author.

## Abbreviation

CoP: Community of Practice
