# Supplementary material for: Training nurses to facilitate and implement CURA in palliative care institutions: development and evaluation of a blended learning program
Source: BMC Palliat Care. 2023 Oct 21;22:158. doi: 10.1186/s12904-023-01284-4 (PMC10590004; doi:10.1186/s12904-023-01284-4)
Supplement: Supplementary file 1 — Additional file 1. [file 12904_2023_1284_MOESM1_ESM.pdf]

CURA

SUPPORT FOR MORALLY DIFFICULT
SITUATIONS IN CARE PRACTICE

Concentrate

- Take a moment to reflect on the situation. Describe this situation briefly.
- What are your doubts concerning good (palliative) care?

→

Unrush

- Identify your initial reaction to the situation and to those involved *(first judgments and emotions)*.
- 'Park' your initial reaction for a while so that you can explore the situation with an open mind.

↓

Reflect

- What is important in this situation?
  - For the patient
  - For those involved *(e.g. family, colleagues, physicians)*
  - For you
- What do laws, protocols, guidelines say?
- What is still unknown or uncertain to you?

→

Act

- What do you find most important in this situation?
- On this basis, what are you going to do?
- How does this relate to what you stand for as a professional?
- Have you gained new insights?
- Have your feelings about the situation changed?

About CURA

What?

CURA is an accessible form of ethics support, which you can use in morally difficult situations in your care practice. Are you uncertain about what is the right thing to do in a difficult situation? Do your ideas about good care differ from others? If so, CURA could be useful.

Why?

CURA can help you in dealing with 'moral distress': the burden that you can experience when doubting if good care is given. CURA can help strengthen your ability to deal with morally difficult situations.

When?

You can use CURA either in an acute situation, or if you want to reflect on the situation at a later moment and consider how you have dealt with it.

With whom?

You can use CURA alone or together, for example during a workshop or team meeting.

Want to know more?

For more information and the manual, send an email to: CURA@amsterdamumc.nl.

Using CURA together

- Plan in advance how much time you want to spend on each one of the four steps.
- For *'Concentrate'* limit yourself to a factual description of the situation *(Who? What? Where?)*.

Individual use

- Consider whom you want to share your views and insights with *(e.g. colleagues or supervisors)*.

Copyright Amsterdam UMC, May 2019. Subject to possible changes.
