# Supplementary material for: Training nurses to facilitate and implement CURA in palliative care institutions: development and evaluation of a blended learning program
Source: BMC Palliat Care. 2023 Oct 21;22:158. doi: 10.1186/s12904-023-01284-4 (PMC10590004; doi:10.1186/s12904-023-01284-4)
Supplement: Supplementary file 2 — Additional file 2. [file 12904_2023_1284_MOESM2_ESM.docx]

# Supplementary files

**Questionnaire: Evaluation of the CURA-ambassador training**

**Topic list for interviews**

**Questionnaire: evaluation of the CURA-ambassador training**

1. Which organization do you work for?

2. What is your profession?

3. Have you successfully completed the training?

4. Did you attend the training sessions in-person or online?

5. I rate the training with a score (1- 10)

6. What did you appreciate in the training?

7. What can be improved in the training?

8. Please answer the following statements about the e-module (1 – 5 Likert scale):

The instructions of the assignments in the e-module were clear

The e-module was technically difficult (such as: logging in, uploading assignments etc.)

The e-module was instructive

The e-module was stimulating

The e-module took too much time

9. Please answer the following statements about the training sessions.

The training sessions were instructive

The training sessions and the e-module were complementary to each other

The trainers were competent

I am sufficiently prepared to use CURA with colleagues.

10. How much time did you spend in total on the training?

11. Is there a topic you would like to learn more about in future masterclasses?

12. Would you like to comment on anything else concerning the training?

**Topic list interviews**

Training structure:

- How did you experience the training sessions? How did you experience the e-module?
- The questionnaires showed that some participants experience the e-module as difficult, as it requires digital skills. What were your experiences in this regard?
- Did you attend the training sessions in-person or online? How did you experience this?
- What is the most important element you learned from the e-module? And during the meeting days?

Content of the training:

- To what extend did the training match your expectations?
- Are there aspects that you missed in the training?
- What have you learned from the e-module? And from the training sessions?

Trainers and knowledge transfer:

- How do you evaluate the quality of the trainers?
- What did you especially appreciate concerning the trainers? What did you appreciate less?
- How do you evaluate the quality of the feedback by the trainers?
